# Improving IQ measurement in intellectual disabilities using true deviation from population norms

**DOI:** 10.1186/1866-1955-6-16

**Published:** 2014-07-08

**Authors:** Stephanie M Sansone, Andrea Schneider, Erika Bickel, Elizabeth Berry-Kravis, Christina Prescott, David Hessl

**Affiliations:** 1 Medical Investigation of Neurodevelopmental Disorders (MIND) Institute, University of California at Davis Medical Center, 2825 50th Street, Sacramento, CA 95817, USA; 2 Departments of Pediatrics, Neurological Sciences, and Biochemistry, Rush University Medical Center, Chicago, IL, USA; 3 Department of Psychiatry and Behavioral Sciences, University of California, Davis, School of Medicine, Sacramento, CA, USA; 4 Department of Pediatrics, University of California, Davis, School of Medicine, Sacramento, CA, USA; 5 Department of Pediatrics, Rush University Medical Center, Chicago, IL, USA

**Keywords:** IQ, Intellectual disability, Autism spectrum disorder, Fragile X syndrome, Cognitive assessment

## Abstract

**Background:**

Intellectual disability (ID) is characterized by global cognitive deficits, yet the very IQ tests used to assess ID have limited range and precision in this population, especially for more impaired individuals.

**Methods:**

We describe the development and validation of a method of raw *z*-score transformation (based on general population norms) that ameliorates floor effects and improves the precision of IQ measurement in ID using the Stanford Binet 5 (SB5) in fragile X syndrome (FXS; n = 106), the leading inherited cause of ID, and in individuals with idiopathic autism spectrum disorder (ASD; n = 205). We compared the distributional characteristics and Q-Q plots from the standardized scores with the deviation *z*-scores. Additionally, we examined the relationship between both scoring methods and multiple criterion measures.

**Results:**

We found evidence that substantial and meaningful variation in cognitive ability on standardized IQ tests among individuals with ID is lost when converting raw scores to standardized scaled, index and IQ scores. Use of the deviation *z-* score method rectifies this problem, and accounts for significant additional variance in criterion validation measures, above and beyond the usual IQ scores. Additionally, individual and group-level cognitive strengths and weaknesses are recovered using deviation scores.

**Conclusion:**

Traditional methods for generating IQ scores in lower functioning individuals with ID are inaccurate and inadequate, leading to erroneously flat profiles. However assessment of cognitive abilities is substantially improved by measuring true deviation in performance from standardization sample norms. This work has important implications for standardized test development, clinical assessment, and research for which IQ is an important measure of interest in individuals with neurodevelopmental disorders and other forms of cognitive impairment.

## Background

The measurement of global intellectual functioning is routinely included in the diagnosis and assessment of individuals with neurodevelopmental disorders. Typically, this is accomplished by utilizing an individually administered standardized test such as one of the Wechsler Scales of Intelligence or the Stanford Binet Intelligence Scales. These assessments yield an intelligence quotient (IQ), a measure of the progress an individual had made in mental or cognitive development compared to same-aged peers [1], and are used to make important decisions in clinical, psychoeducational, and research arenas. This score has an impact on the allocation of educational and clinical services, monitoring developmental progress or decline, and can significantly affect the results and interpretation of research. Furthermore, these measures are especially critical for identification and diagnosis of individuals with intellectual disability (ID). However, the very tests designed to capture intellectual capacity have limited precision in this population.

ID is a disability present during early development (before 18 years of age) and characterized by significant limitations in both intellectual functioning and adaptive behavior (American Association of Intellectual and Developmental Disorders, http://www.aaidd.org). While IQ is not the only means of evaluating mental capacity for reasoning, learning, and problem solving, it is the currently recommended description of general cognitive ability in the *Diagnostic and Statistical Manual of Mental Disorders* (5th edition; DSM-5) [2]. An IQ of around 70 or below, obtained using a standardized, individually administered intelligence test indicates significant deficits in cognitive functioning.

Researchers have long acknowledged the issues of using tests that are imprecise and less reliable in the lower ability ranges of individuals diagnosed with FXS [3]. Most intelligence tests do not measure IQ below 40 (for example, Wechsler Scales, Stanford Binet Intelligence Scales, Kaufman Assessment Battery for Children, Universal Non-Verbal Intelligence Test) and their ability to accurately and reliably measure intellectual functioning below the mild ID range is limited, especially when flooring occurs. When test developers limit the lower range of item/task difficulty, they minimize test sensitivity among individuals in this range of functioning. Also, the majority of IQ tests exclude, or do not actively include individuals with ID in their standardization samples, rather using these individuals exclusively for separate validation studies (for example, Differential Ability Scales, Second Edition; Wechsler Scales; Leiter International Performance Scale, Revised). This is likely to further limit sensitivity and validity of lower scores, as there are few or no individuals in the standardization sample with ID to represent their ability level and pattern of performance. Some individuals with a neurodevelopmental disorder are likely to score at the extreme low end of a test’s population-normed distribution, contributing to flooring effects and an inadequate assessment and profile of the person’s true abilities. These types of measurement issues can be especially problematic in research. One of the expectations of many statistical methods employed in research assumes the data approximate a normal distribution. Intelligence tests with large floor effects typically have reduced range and variability, and create positively skewed data that produce significant deviations from normality, in samples of individuals with ID (for examples see references [4,5]). However, it should be noted that some test publishers have made revisions to IQ tests allowing a somewhat lower floor. For example, the Leiter International Performance Scale, Revised and the Differential Ability Scales, Second Edition each have a lower IQ limit of 30.

The floor effects of intelligence testing are occasionally acknowledged in the literature, but rarely addressed. Furthermore, the few attempts to resolve this issue are inconsistent and often study sample specific. For example, when Couzens *et al*. [6] found that 37% of their sample of individuals with Down syndrome scored at the floor on the Stanford Binet, Fourth Edition, they reported that using the Mean Age Equivalent (MAE) score helped reveal some of the variability in performance masked by the floor effect. However, they also mentioned that using the MAE score continued to be problematic because it leads to inaccurate estimation of many skills, and can only be interpreted relative to the individual’s chronological age. Whitaker and colleagues [5,7] discovered floor effects on multiple versions of the Wechsler Intelligence Scale for Children (WISC) and they recommended extrapolating the relationship between subtest raw scores and scaled scores below a scaled score of one. An issue with this approach is the scale is no longer uniform across all standardized and extrapolated scores. Still other researchers have addressed the issue by using re-standardized raw scores based on their sample specific statistics [8], but this prevents comparison of results across study samples. Even worse, some studies have excluded those who score at the floor from their statistical analyses (for example [9]) limiting the generalizability of their findings and possibly biasing study findings. Furthermore, evaluations of these revised scoring methods have relied mostly on graphical or descriptive analyses rather than comparing the psychometric properties of the original standardized scores to new scoring methods.

Recently, we and others [4] began to develop and evaluate a solution to address the flooring problem on the WISC-III. In a sample of 217 children with fragile X syndrome (FXS), all subtests demonstrated significant skewing and flooring for a high proportion of individuals. For example, 94% of the males in the study obtained floored scaled scores on the arithmetic subtest. With permission from the publisher, raw score descriptive statistics from the original standardization sample were used to calculate new normalized scores (heretofore referred to as ‘deviation’ scores for clarity) by applying a *z*-score transformation to subtest raw scores for each individual. Unlike standardized scores, the deviation scores extended downward to more than three standard deviations below the mean. These deviation scores were normally distributed and no longer demonstrated flooring problems. To validate this new scoring method, we established that the deviation scores were more strongly correlated with adaptive behavior (Vineland Adaptive Behavior Scales) and with a genetic measure specific to FXS (*FMR1* protein, FMRP) compared to the traditional IQ scores. Unfortunately, the Wechsler use agreement expired and could not be renewed, preventing future use of the method with the WISC or other Wechsler Scales.

Therefore, based on the work described by Hessl and colleagues [4], here we extend this method to the Stanford Binet, Fifth Edition (SB5), with permission from the test publisher and authors, in a large sample of individuals with a confirmed diagnosis of autism spectrum disorder (ASD) or FXS. Our goal was to replicate previous findings [4] in a similar sample using a different commonly utilized intelligence test with greater developmental range, and to extend the method to idiopathic autism, a much more common condition associated with ID. In particular, compared to previous editions, and to the Wechsler Scales, the SB5 includes enhanced non-verbal content requiring only minimal or no verbal response, increased breadth of items/tasks at a very low level of developmental functioning, and covers a wider age range (2 to 85+ years).

FXS is the leading known cause of inherited ID and recent estimates of the frequency of the full mutation are as high as one in every 2,500 births [10]. FXS is caused by the expansion of a trinucleotide repeat sequence, cytosine-guanine-guanine (CGG), in the promoter region of the Fragile X Mental Retardation 1 gene (*FMR1*) on the long arm of the X chromosome at Xq27.3. Expansions greater than 200 repeats lead to hypermethylation of the gene and absence or significant deficit of FMRP, an mRNA binding protein that regulates (predominantly inhibiting) the translation of many neuronal messages coding for key synaptic proteins. FMRP is necessary for normal brain development including dendritic arborization and synaptic plasticity [11-13]. Many researchers have and continue to examine the impact of gene function, neurodevelopment, and environment on cognitive functioning in FXS with the aim of better understanding the developmental trajectory and endophenotypic variation, in order to develop effective interventions. The specific genetic etiology of FXS has been well defined, giving researchers a unique opportunity to better understand the sensitivity of intelligence testing, and more specifically this revised scoring method in an ID population.

Additionally, we chose to include a second sample from a more heterogeneous and prevalent ID population of individuals diagnosed with ASD from the Autism Genetic Resource Exchange (AGRE) data repository. ASD is a neurodevelopmental disorder characterized by deficits in social interactions and communication, and restricted, repetitive patterns of behavior, interests or activities. Previously, researchers have used tests of cognitive functioning to predict the severity of autism symptoms and as an outcome measure of early intervention programs for ASD (for examples see [14,15]). Given the importance of intelligence tests in this research, the fact that a high proportion of individuals with ASD have comorbid ID (see [16] for review), and the increasing numbers of children and youth with ASD in the population [17], we reasoned that it may be especially important to examine the accuracy of IQ scores in this population.

The present study had three aims: (a) to investigate the effect of the aforementioned deviation scoring method on various distributional and psychometric characteristics of the SB5 (b) to assess whether the scoring method improves the test’s concurrent validity, and (c) to determine how the scoring method affects the interpretation of relative strengths and weaknesses in an individual’s cognitive ability profile, as well as in group-level cognitive profiles of ASD and FXS. A long-term goal of this program of research is to provide critical cognitive psychometric data from a large sample of individuals with ID to support the future development of more precise IQ tests and assessment methods for these individuals.

## Methods

### Sample

Our analyses were carried out on 311 individuals with a diagnosis of FXS or ASD ranging in age from 3 to 38 years (54 females, mean age = 10.61 ± 4.57 years; 257 males, mean age = 11.93 ± 5.95 years). The ethnic distribution was 63.41% Caucasian, 16.1% Hispanic, 4.5% African American, 4.8% Asian, 0.3% Native American/Pacific Islander, 6.1% Multi-ethnic and 4.8% unknown or not reported. Individuals included in these analyses had an IQ < 70 and a score of 1 (a floored subtest score) on at least one of the ten subtests on the SB5. Full Scale Intelligence Quotient (FSIQ) for the total sample ranged from 40 to 69 (mean 48.73, SD 9.13). Sample data was brought together from three sources. Individuals with FXS were recruited through the fragile X research and clinical programs at the UC Davis MIND Institute (n = 68) and the Rush University Medical Center (n = 38). FXS diagnosis was confirmed using southern blot analyses which were performed according to procedures described by Taylor and colleagues [18].

The third source was an archival dataset provided through AGRE. Families with at least one member who had received a diagnosis of autism from a family physician or autism specialist were recruited during community events and using web-based media. Following registration and consent, qualified staff administered a battery of assessments and confirmed a clinical diagnosis of an ASD using the Autism Diagnostic Interview-Revised (ADI-R; [19]) and the Autism Diagnostic Observation Schedule (ADOS; [20]). Of the 1,028 total cases available from the AGRE dataset, 791 had a valid IQ score and met criteria for ASD based on the ADI and ADOS. Two hundred and five of these (25.9%) had an IQ less than 70 and at least one floored score for inclusion in our analytic sample. The parents/legal guardians of all participants provided written consent, and participants provided assent when possible, according to protocols approved by the Institutional Review Boards at UC Davis, Rush University, the Western Institutional Review Board, and UC Los Angeles.

### Measures

Stanford Binet Intelligence Scales, Fifth Edition (SB5; [21]). The SB5 is a standardized test of intellectual aptitude for children and adults between ages 2 to 85 years. The fifth edition was developed and structured based on the Cattell-Horn-Carroll (CHC; [22]) theory of intelligence. The CHC model conceptualizes intelligence as having a hierarchical structure with three levels: narrow abilities at the lowest level, broad cognitive abilities in the middle and a general measure of cognitive ability (g) at the highest level.

The SB5 provides a general ability score reported as the FSIQ, and five index scores that measure the broad cognitive concepts of Fluid Reasoning (FR), Knowledge (KN), Quantitative Reasoning (QR), Visual Spatial Processing (VS), and Working Memory (WM). These five indices are measured across two broad response domains, Verbal (VIQ) and Non-verbal (NVIQ), in total providing ten subtest scores. These subtests (with the exception of two routing subtests) are measured across five or six testlets that vary in level of difficulty. Each testlet has a range of possible raw scores from 0 to 6 and is made up of 3 to 6 items. Raw scores from testlets within the same subtest are summed together and then transformed into a scaled score with a mean of 10 and standard deviation of 3 based on SB5 normative data. Normative data is based on a standardization sample of 4,800 individuals stratified by age, sex, race/ethnic group, geographical region, and socio-economic status. The subtest scaled scores are then combined and translated into index scores and the three intelligence quotients (VIQ, NVIQ, and FSIQ). The SB5 introduced a new scoring method for deriving an extended IQ score (EXIQ) that broadened the range of scores from 40 to 160 to 10 to 225. For EXIQ, using the one-parameter Rasch model, the total raw scores were converted into a change sensitive score (CSS). Using traditional methods, norms for the CSS score were calculated for all 30 age groups and then re-scaled to the IQ metric.

### Criterion measures

A large portion of the sample completed additional criterion measures. Some individuals from both diagnostic groups had data from the Vineland Adaptive Behavior Scales, Second Edition (n = 185 with ASD, n = 94 with FXS). Only individuals from the AGRE dataset had available data from the Peabody Picture Vocabulary Test (n = 184) and Raven’s Colored Progressive Matrices (n = 172).

Vineland Adaptive Behavior Scales, Second Edition (VABS-II; [23])*.* The VABS-II is a widely used tool for assessing personal and social skills needed for everyday living. It is a semi-structured informant interview for assessing strengths and weaknesses of individuals from birth through 18 years, 11 months or low-functioning adults. Part of the utility of this measure is the ability to gain accurate reporting from a responder who is familiar with a person’s behavior and skills in everyday life. Adaptive behavior is measured in four to five domains: Communication (receptive, expressive and written), Daily Living Skills (personal, domestic, and community), Socialization (interpersonal relationships, play and leisure time, and coping skills), and Motor Skills (gross motor and fine motor; completed only for the youngest children). The Adaptive Behavior Composite and Communication standard scores (ranging from 20 to 160) were used as criterion measures for the FSIQ and VIQ, respectively.

Peabody Picture Vocabulary Test, Third Edition (PPVT-3; [24])*.* The PPVT is a norm-referenced assessment of receptive vocabulary. The examinee is required to choose from a set of four pictures, the one that best relates to the vocabulary word said aloud by the examiner. Raw scores are converted to age-adjusted standardized scores, ranging from 20 to 160. The PPVT-3 was used to examine the validity of VIQ based on both scoring methods.

Raven’s Colored Progressive Matrices (RCPM; [25]). The RCPM is a standardized test of non-verbal intelligence. Each item is a colored pattern with a missing portion and the examinee is asked to choose the missing elements from a group of six possible options. Raw scores are converted to an IQ score, with a range of 28 to 148 depending on age band. The RCPM is most similar to tasks used on the non-verbal Fluid Reasoning subtest (NVFR) from the SB5 and was used as a criterion measure for this subtest.

### Statistical methods

Deviation scores. The deviation scores were derived using an age-dependent (within each population age band) *z*-score transformation as follows. Descriptive statistics (means and standard deviations) of subtest raw scores for each age band from the SB5 standardization sample were obtained with written permission from the SB5 publisher, PRO-ED, Inc. (Austin, TX, USA) for the purposes of this study. The deviation score for individual *i* falling into the *j*th age band is:

$$z_{\mathrm{ij}}=\frac{r_{\mathrm{ij}}-\mu_{j}}{\sigma_{j}}$$

where *r*_*ij*_ is the subtest raw score, μ_j_ and σ_j_ denote the mean and standard deviation from the corresponding age band and subtest from the standardization sample. We created a function using the R program [26] that automated the process of converting raw to normalized scores. Additionally, to make comparison between the standardized scores and deviation score more intuitive we converted the scale of the deviation *z-*score from a mean of 0 and standard deviation of 1 to the scale used for standardized subtest scores with a mean of 10 and standard deviation of 3.

For example, consider a 10-year-old participant obtaining a subtest raw score of 10 on the NVFR subtest. In the standardization sample, for children 10 years old, the NVFR mean raw score is 24.31 and the standard deviation is 3.37. Therefore, the child’s deviation *z-*score is (10 – 24.31)/3.37 = −4.25, or 4.25 standard deviations below the mean for their same age-peers in the SB5 normative sample. Now, when rescaling the *z-*score to mimic the scale of the standardized scores we simply multiply the *z-*score by 3 and add 10, so the deviation score is (−4.25*3) + 10 = −2.75. Using the original standardized scoring method, the individual has a floored subtest score equal to one, which is three standard deviations below the mean for their age group and the lowest score the test will allow. However, this score is an inflated estimate of their true ability when compared to the −2.75 score from the deviation scoring method and represents a raw score that is more than four standard deviations below the mean. In other words, the skill level of any individual who receives a negative score is more than 3.33 standard deviations below the mean for their age group.

We also calculated each participant’s mean deviation scores and rescaled the mean *z*-score with a mean of 100 (SD = 15) to indicate the overall deviation from the normative sample across subtests, analogous to the subtest standardized score combinations used to generate the five broad index scores as well as the NVIQ, VIQ and FSIQ.

### Analysis

For both scoring methods we compared the distributional characteristics, including mean, standard deviation, range, skew, and kurtosis. Quantile-quantile normal probability plots of the NVIQ, VIQ, and FSIQ, provide a visual description of how close (or far) these scores are from the normal distribution. The normality of the EXIQ scores was also examined. In these plots the quantiles (percentiles) from our sample data are plotted against the quantiles from a theoretical normal distribution and ideally form a diagonal straight line. The observed data points are sorted in ascending order and paired with a second set of theoretically expected values based on the standard normal distribution. A scatter plot is created based on these two sets of values. The closer the correlation between these two distributions is to 1.0 (the reference line), the closer the sample data is to a normal distribution. Departures from a straight line indicate a departure from normality. The addition of 95% confidence intervals aids in the interpretation of whether a small deviation from the reference line can still be interpreted as being consistent with a normal distribution.

To examine the relative contribution of the deviation scores compared to the standardized scoring method when predicting the criterion measures, we used methods similar to those used in studies examining incremental validity. Following methods discussed in [27], hierarchical linear regression was used to estimate the proportion of variance in the criterion variables associated with the new deviation scoring method above that associated with the traditional standardized subtest, IQ, or index scores and to test whether any increase in predictive efficacy was significant. In these analyses, any shared variance between the original standardized scores and the new deviation scores is attributed to the standardized scores only, making this a rigorous test of the additional information provided by the new method. Analyses were performed in R [26].

When considering how the usual and new deviation scoring methods might impact clinical or educational decisions, we used two cases to illustrate differences in the interpretation of individual profiles, including their strengths and weaknesses on various subtests. Finally, we plotted and compared the standardized versus deviation subtest scores separately for the ASD and FXS samples to examine the cognitive ability profiles of relative strengths and weaknesses of these two diagnostic groups.

## Results

As expected, the standardized scores from the subtests exhibited pervasive flooring effects. A summary of the flooring effects resulting from the use of standard scores is shown in Table 1. The percentage of participants with floored standard scores on subtests ranged from 43.4% (non-verbal Quantitative Reasoning) to 83% (verbal Working Memory). The range of raw scores corresponding to a floored score of one extended from 0 to 9 to as large as 0 to 24, demonstrating that conversion of raw scores to standard scores masks a large portion of variability in performance. Table 2 displays the percentage of floored scores across all subtests by IQ band. About a fifth of all subtest scores were at the floor even for those with IQs in the 60s.

**Table 1:** Descriptive statistics for subscales scored using standardized and deviation scores

**Subtests**	**Percent of standard score of 1 (floored)**	**Percent of raw score of 0**	**Range of floored raw scores**	**Scoring method**	**Mean (SD)**	**Median**	**Range**	**Skew**	**Kurtosis**
			Non-verbal subtests						
Fluid reasoning	57.6%	1.0%	0 to 17	Standard	2.97 (2.93)	1.00	12.00	1.44	1.18
				Deviation	0.24 (5.97)	0.55	29.37	−0.17	0.68
Knowledge	54.3%	3.2%	0 to 13	Standard	2.26 (1.80)	1.00	8.00	1.44	1.26
				Deviation	−0.48 (4.51)	0.82	21.27	−0.32	−0.84
Quantitative reasoning	58.5%	11.9%	0 to 13	Standard	2.47 (2.27)	1.00	11.00	1.59	1.86
				Deviation	−0.98 (5.67)	0.25	24.59	−0.25	−0.93
Visual spatial	43.4%	0.3%	0 to 13	Standard	3.87 (3.37)	3.00	14.00	0.99	0.02
				Deviation	2.29 (5.24)	2.50	28.30	−0.22	−0.28
Working memory	56.5%	0.6%	0 to 13	Standard	2.63 (2.40)	1.00	11.00	1.49	1.47
				Deviation	0.15 (5.09)	0.46	23.89	−0.11	−0.79
			Verbal subtests						
Fluid reasoning	76.0%	47.1%	0 to 9	Standard	1.75 (1.62)	1.00	8.00	2.44	5.53
				Deviation	−1.68 (4.65)	−1.87	18.66	0.19	−1.00
Knowledge	74.0%	2.9%	0 to 24	Standard	1.59 (1.23)	1.00	7.00	2.44	5.85
				Deviation	−1.27 (3.94)	−0.62	21.41	−0.56	0.07
Quantitative reasoning	50.0%	13.3%	0 to 10	Standard	2.67 (2.12)	2.00	9.00	1.17	0.53
				Deviation	0.26 (4.60)	0.72	21.96	−0.60	−0.13
Visual spatial	73.4%	2.9%	0 to 13	Standard	1.71 (1.46)	1.00	9.00	2.39	5.88
				Deviation	−1.67 (4.19)	−1.49	22.43	−0.10	−0.60
Working memory	83.8%	16.2%	0 to 13	Standard	1.49 (1.35)	1.00	8.00	3.19	10.10
				Deviation	−4.23 (5.98)	−3.60	31.12	−0.49	0.24

**Table 2:** Percent of subtest scores at the floor by Full Scale Intelligence Quotient (IQ) band

**Level of IQ**	**n**	**Floored scores**
40s	195	82.6%
50s	58	37.1%
60s	58	20.0%

**Figure 1:**
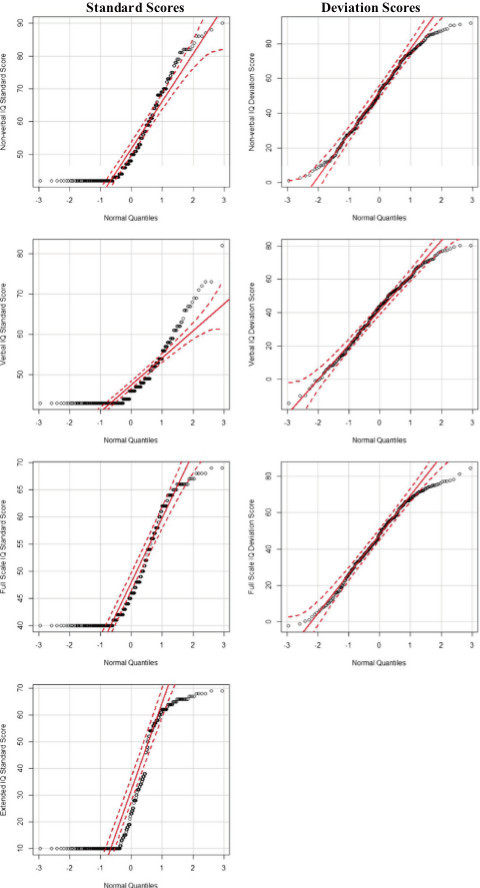
**Quantile-quantile (Q-Q) plot of the standardized and deviation intelligence quotient (IQ scores).** Open circles represent the actual data points. The solid, diagonal red line is the reference line for the expected, normal distribution. The dotted lines represent the upper and lower bounds of the 95% confidence interval. EXIQ, Extended IQ; FSIQ, Full Scale IQ; NVIQ, Non-verbal IQ; VIQ, Verbal IQ. For the EXIQ plot, standard Full Scale IQ is shown above IQ = 40 for presentation and plotting purposes.

Additionally, the descriptive statistics for each subtest using standard scores and deviation scores are shown in Table 1. The distribution characteristics for the deviation scoring method were superior overall compared to the original standard scores. As expected, the means for all deviation scores were lower and the ranges were larger compared to the standard scores. The absolute values for skew were smaller for every deviation subtest score compared to the standardized scores. The same was also true for kurtosis, except for non-verbal visual spatial processing. The distributional characteristics of the censored and discrete standard scores are less desirable for statistical analysis compared to the revised deviation scores.These findings are further corroborated by examining the quantile-quantile normal plots created for the VIQ, NVIQ, EXIQ and FSIQ, displayed in Figure 1. The plots of the deviation scoring method display a more normal distribution. With the standardized scores, many of the points fall outside of the 95% confidence limit at both ends of the distribution and especially for those with a floored IQ score of 40. Some of the deviations from normality we see in the higher IQ range could be due to our imposed FSIQ upper limit of 69. Even for EXIQ scores, which have a wider range than the standardized FSIQ scores, many points deviate from the reference line starting around 40 and especially around scores of 10 (the EXIQ floor). The floored scores that contribute to the non-normality are ameliorated by the deviation scoring method, with the data points for all three IQ scores falling on or near the references line and within the 95% confidence interval in the lower IQ range. Since the focus of the method described here is to help correct for flooring effects, we would not expect it to correct the small deviations from normality in the higher tail of the IQ range used for this study.

### Criterion measures

**Figure 2:**
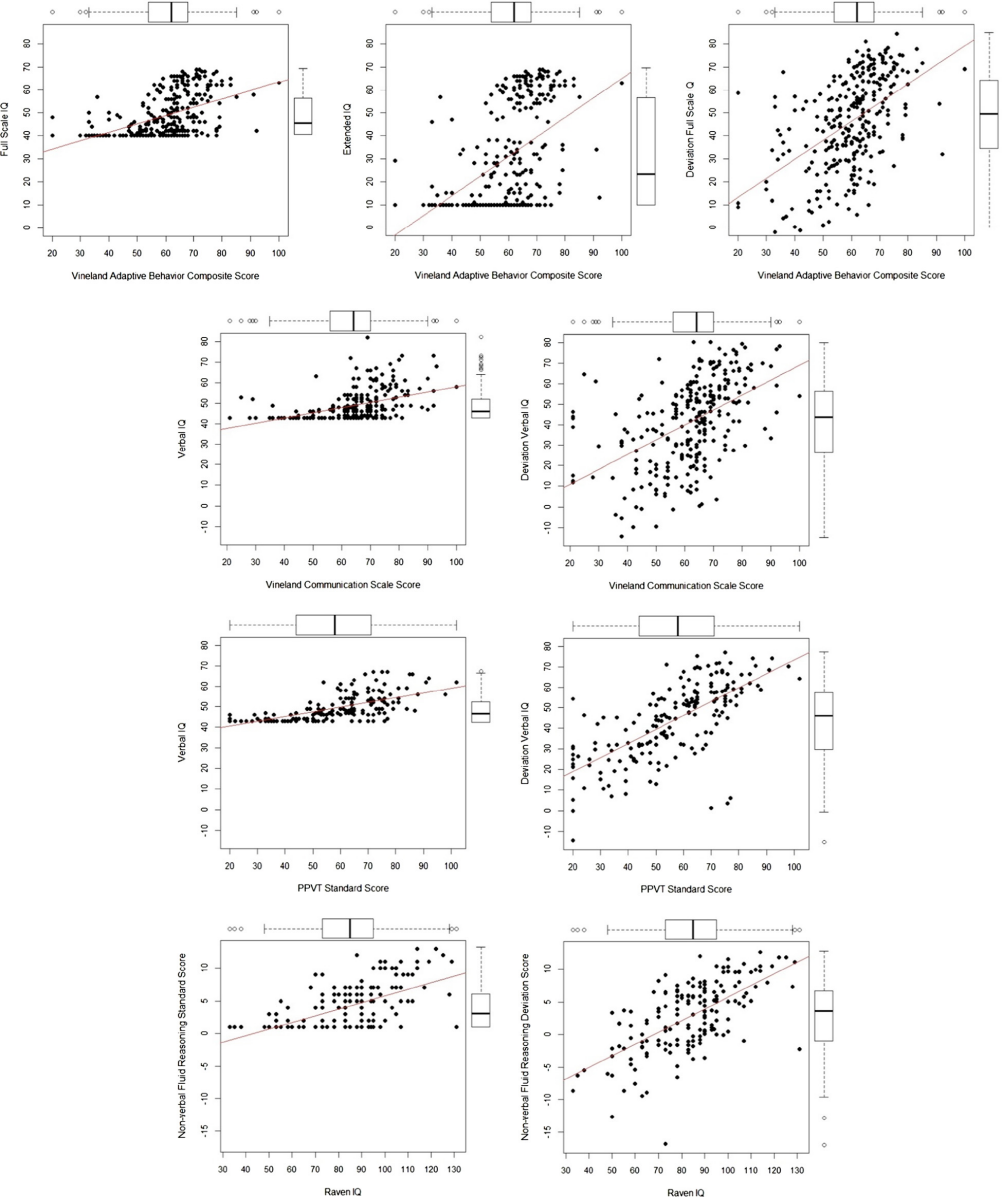
**Bivariate association of deviation and standardized IQ scores with their corresponding criterion measures with marginal boxplots.** EXIQ, Extended IQ; FSIQ, Full Scale IQ; NVIQ, Non-verbal IQ; PPVT, Peabody Picture Vocabulary Test; VABC, Vineland Adaptive Behavior Composite; VIQ, Verbal IQ.

Scatter plots in Figure 2 display the relationship between the deviation IQ scores and the standardized IQ scores with their respective criterion measures. As was observed with the subtests, IQ was floored for a large portion of our sample (27.7% for NVIQ and FSIQ, 39.5% for VIQ). Additionally, examination of the marginal boxplots in Figure 2 shows that the original standardized scoring method is more likely to falsely identify outliers compared to the deviation method.

Table 3 displays the results from hierarchical regression analyses used to examine whether the deviation scoring method predicts additional variance in the criterion measures above and beyond the standardized scores. VIQ was a significant predictor of both the VABS-II communication score and the PPVT, however the deviation VIQ predicted significant additional variance (Δ*R*^2^ = 0.03, *P* < 0.001 and Δ*R*^2^ = 0.06, *P* < 0.001, respectively).

**Table 3:** Summary of hierarchical regression analyses

**Criterion score**	**Step**	**Scale**	***r***	**Final** ***β***	***R***^**2**^	**Δ** ***R***^**2**^
Vineland composite	1	FSIQ	.48	.19	.23	
	2	+ FSIQ z-score	.51	.35	.26	.03^a^
	1	EXIQ	.48	.13	.22	
(n = 279)						
	2	+ FSIQ z-score	.51	.39	.25	.03^a^
Vineland communication (n = 279)	1	VIQ	.49	.27	.24	
	2	+ VIQ z-score	.50	.29	.27	.03^a^
PPVT (n = 184)	1	VIQ	.67	.32	.45	
	2	+ VIQ z-score	.69	.43	.51	.06^a^
Raven (n = 173)	1	NVFRSS	.57	.07	.32	
	2	+ NVFR z-score	63	.58	.40	.08^a^

^a^*P* < .001; *r* = correlation between predictor and criterion variable.

EXIQ, Extended IQ; FSIQ, Full Scale IQ; NVFRSS, Non-verbal Fluid Reasoning Standardized *S*core; VIQ, Verbal IQ; PPVT, Peabody Picture Vocabulary Test.

We used the same model to test both the FSIQ and EXIQ to examine whether the deviation IQ score would predict additional variance in the VABC score above what could be accounted for with the FSIQ or EXIQ. The deviation IQ score incrementally predicted a significant amount of the variance in the VABC score above the standardized FSIQ and EXIQ (Δ*R*^2^ = 0.03, *P* < 0.001 and Δ*R*^2^ = 0.03, *P* < 0.001, respectively). Furthermore, FSIQ (*β* = 0.48, *P* < 0.001) and EXIQ (*β* = 0.47, *P* < 0.001) were significant upon entry but failed to retain their predictive power (*β* = 0.19, *P* = 0.06 and *β* = 0.13, *P* = 0.22, respectively) when the deviation IQ was added to the model (*β* = 0.35, *P* < 0.001 with FSIQ and *β* = 0.39, *P* < 0.001 with EXIQ).

Finally, the deviation scoring methods contributed an additional 8% of variance in predicting the RCPM over the standardized score on the NVFR subtest (Δ*R*^2^ = 0.08, *P* < 0.001). Again, the inclusion of the deviation score significantly reduced the influence of the standardized scores from *β* = 0.57 (*P* < 0.001) to *β* = 0.09 (*P* = 0.437) when the deviation NVFR score was added (*β* = 0.55, *P* < 0.001).

### Examples of cognitive profile differences based on traditional standard and deviation scoring methods

**Figure 3:**
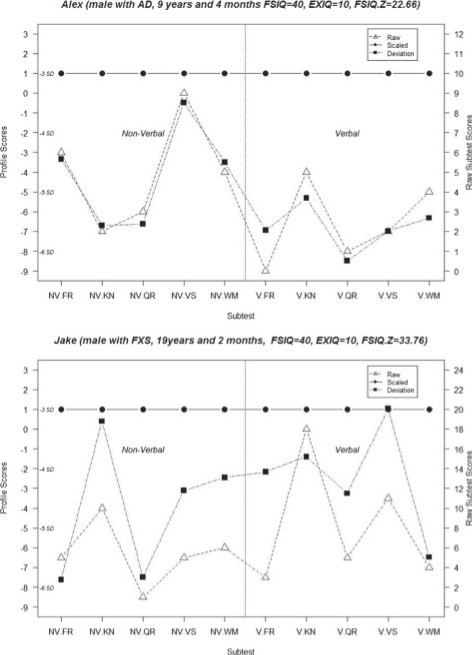
**Subtest raw scores (open triangles), standardized scores (closed circles), and deviation (z) scores (closed squares) for two case examples.** FR, fluid reasoning; KN, knowledge; NV, non-verbal; QR, quantitative reasoning; V, verbal; VS, visual spatial processing; WM, working memory.

Figure 3 provides two case examples of how the practical application of this scoring method may improve a clinician’s understanding of a patient’s cognitive strengths and weaknesses compared to the traditional standardized scoring method. ‘Alex’ is a nine-year-old male diagnosed with autism. His cognitive assessment yielded a FSIQ of 40, EXIQ of 10, and VABC score of 61. As can be seen in panel (A), Alex received a standard score of 1 across all subtests producing the horizontal line with no variability and possibly leading to the conclusion that he is equally affected across all domains on the SB5. In contrast, we see that his performance on nine of the ten subtests are actually more than four standard deviations below the mean and he shows a larger deficit on many of the verbal subtests relative to their non-verbal equivalents, especially in visual spatial processing.‘Jake’ is a 19-year-old male diagnosed with FXS, who received a FSIQ of 40, EXIQ of 10, and a VABC score of 32. While Jake and Alex had identical FSIQ, EXIQ, and standardized subtest scores, their deviation score profiles are substantially different. In panel (B) (Figure 3), despite a flat profile of standardized scores, deviation subtest scores reveal that non-verbal fluid reasoning and quantitative reasoning are areas of relative weakness while he performs better on non-verbal knowledge and verbal visual spatial processing.

### Stanford Binet Index profiles based on standardized scores compared to deviation scores by diagnosis

**Figure 4:**
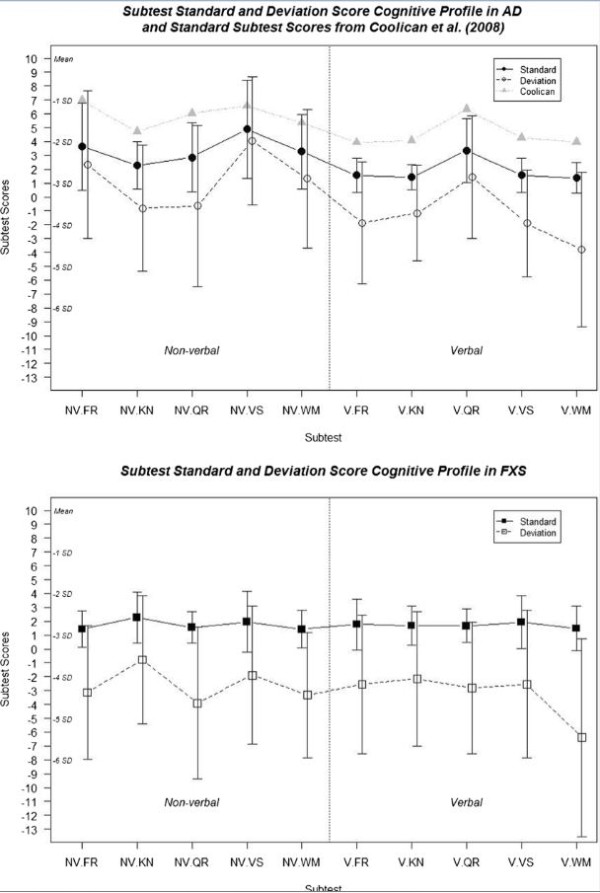
**Profile of standardized and deviation (z) subtest scores by diagnosis group (mean ± SD).** FR, fluid reasoning; KN, knowledge; NV, non-verbal; QR, quantitative reasoning; V, verbal; VS, visual spatial processing; WM, working memory.

In addition to examining the relative cognitive strengths and weaknesses in two case examples, we also examined how the typical standard score versus deviation score methods affect representation of ASD and FXS cognitive profiles. In a recent report, Coolican and colleagues [28] examined the cognitive profiles of children with ASD on the SB5. Figure 4 has a visual comparison of their sub-sample of individuals with autistic disorder (n = 32) with a sub-sample from our own data (n = 163) that covered the same age range (3 years and 16 years, 10 months), diagnosis (met criteria for autism on both the ADI-R and the ADOS), and similar gender ratio (more than three quarters male). Additionally, Figure 4 displays the cognitive profiles of a sub-sample of individuals with FXS with the same restricted age range.

The flooring effects from the standardized scores have a flattening effect on the profile for both individuals with ASD and FXS. This was especially true in the FXS group, which had a much larger portion of individuals who scored at the floor compared to those with ASD. This finding in the FXS group is not unique to this study or to the SB5. Fisch [29] found a relatively flat profile using the standard index/area scores from the Stanford Binet, Fourth Edition among females with either the premutation or full mutation. Additionally, Backes and colleagues [30] reported similar findings in a sample of boys with FXS using the Kaufman Assessment Battery for Children. Remarkably, the pattern of standard scores from our ASD sub-sample almost perfectly replicated those reported by Coolican and others [28]. The subtest scores reported in that study are higher than those found here, probably a product of our IQ restriction of less than 70. The similarity of cognitive profiles suggests that our results are not merely a reflection of a unique sample, and provides additional validation that other studies would benefit from these scoring methods. Across both groups the subtest with the largest difference between the standardized and deviation scores was verbal working memory; it also had the largest proportion of individuals with ASD and FXS (both greater than 80%) who received a standard score of 1. Overall, the ASD group shows relative strengths in non-verbal visual spatial reasoning and verbal quantitative reasoning, with perhaps relative weakness in verbal working memory. The FXS group also showed a relative weakness in verbal working memory however non-verbal knowledge was a relative strength.

## Discussion

The results of the present study provide evidence of the extent of floor effect limitations on IQ assessments in individuals with intellectual disabilities, and highlight a validated method for improving measurement sensitivity in a relatively large proportion of these individuals. As might be expected, a large percentage of standardized subtest scores (82.6%) received a score of one among individuals with IQs in the 40s. This proportion remained relatively high in the 50s and 60s range at 37% and 20% respectively. These results are similar to those reported by Whitaker and Wood [5], who found that 31.5% and 15.3% of subtests had a scaled score of 1 on the WISC-III and the WAIS-III, respectively.

Compared to the traditional standardized scores, the deviation scores were better distributed and more strongly correlated with measures of adaptive behavior, vocabulary and non-verbal reasoning. Even when we consider that FSIQ, NVIQ, and VIQ scores are highly correlated with their corresponding deviation scores (.77 to .87), these two methods should not be considered equivalent. It should be mentioned that collinearity between the standardized scores and deviation scores could potentially produce unstable and possibly biased beta weights in our incremental validity analysis. However, considering multiple indices, like correlations with criterion measures and Δ*R*^2^, reduces issues with interpretability. Considering all these indices from the results of the hierarchical regression analyses, the deviation scores were better predictors of other cognitive and adaptive behavior measures and accounted for additional variance in these measures over and above the standardized IQ scores. It is likely that the deviation scoring method captures the same information as the traditional scoring method as well as significant and clinically meaningful additional variation from the raw scores that is otherwise lost when they are converted to standard scores.

The improved sensitivity of the deviation scores derived for the study also appear to reflect meaningful individual variation and group-level variation in cognitive abilities, which are masked by the usual IQ scoring method. The scoring method described here expands on previous attempts to address flooring effects on IQ measures by providing the distributional properties of the revised scores and testing their validity. This method has the additional benefit of not being study sample specific. In other words, the method is appropriate for the majority of individuals with ID (of any etiology) who can complete a valid test administration, obtain raw scores above zero (reflecting an ability to understand and engage in test items) and have multiple floored subtest scores. In addition, we wish to emphasize that the EXIQ, with a lower limit of 10, does not ameliorate floor effects (29.0% of ASD, 49.5% of FXS and 35.7% overall obtained this score despite substantially higher cognitive and adaptive functioning), and thus does not appear to provide a valid estimate of intellectual functioning in this low range.

The results of this study have important implications for current and future research. IQ is utilized in many fields as an outcome of interest, an independent variable, or a tool for group matching. For example, when comparing individuals with FXS only to a sample of those with FXS and autism, Lewis and colleagues [31] created two gender and cognitive ability matched groups to limit confounding effects. Since their entire FXS + ASD group had NVIQ scores at the floor (≤ 36), they only selected a sub-sample of individuals from the FXS group who also scored at the floor. Consequently, both groups had an average NVIQ of 36 with a standard deviation of 0, preventing adequate matching on this variable. We suspect that many published studies including participants with ID to date that have relied on IQ scores for matching or for use as a critical variable of interest were negatively impacted by floor effects and lack of measurement sensitivity. Indeed, the IQ and standard score flooring problems may be pervasive across test instruments used in neurodevelopmental disorders. For example, in a sample of 243 young children with ASD (not restricted to having an ID) assessed with the Mullen Scales of Early Learning [32] at the University of Washington Autism Center, 72 (30%) obtained a floored composite score of 49 and the distribution of scores was non-normally distributed and highly skewed (Jeffrey Munson, Geraldine Dawson, and Sally Rogers, Personal Communication).

It is important to note that the age bands used for calculation of deviation scores, provided by the publisher, each had a span of one year, whereas the age bands used for standardized score calculation in the Stanford Binet Examiner’s manual are in three month increments (for example, ten years, zero months through ten years, three months). We feel that this difference is unlikely to have affected the interpretation of the scoring methods. Additionally, caution should be used when interpreting the group level subtest profiles for ASD and FXS. The groups used to create these profiles included individuals from a very large age span. These profiles may look very different at different stages of development.

There have been some improvements in the normative sampling of lower functioning school-aged individuals in the development of cognitive tests; specifically 5% of the normative sample in the SB5 included individuals enrolled in special education that were mainstreamed and spending more than 50% of their school day in a regular classroom. However, this equates to only approximately ten children per sub-sample. It would be worthwhile if, in future standardization studies, a larger number of individuals representative of a more widespread span of functioning in the lower range were included in these samples.

Clinical studies using IQ, or other standardized test scores with pervasive floor effects, to track development or response to intervention may be substantially improved by use of the deviation scoring method for ID. It will become increasingly important to use reliable and sensitive cognitive tests for tracking changes especially given the increasing popularity of interventions that specifically target cognitive development. Publication of test standardization norms by age band (means and standard deviations of raw scores) would make it possible for researchers and clinicians to calculate deviation scores as described here. To facilitate use of the deviation score method described here, PRO-ED, Inc. has posted the SB5 raw score standardization data (means and standard deviations for each subtest by age band; http://www.proedinc.com/Downloads/13290SB-5AgeMeanSDraws.pdf). However, it should be noted that widespread acceptance and application of this method may require further empirical evaluation by the research community, clinical entities, and test development experts.

## Conclusions

In conclusion, our present findings suggest that use of scores that reflect true deviation in performance from general population norms can help ameliorate pervasive flooring problems and more sensitively capture the cognitive abilities of individuals in the lower intellectual functioning range.

## Abbreviations

ADI-R: Autism Diagnostic Interview-Revised; ADOS: Autism Diagnostic Observation Schedule; AGRE: Autism Genetic Resource Exchange; ASD: autism spectrum disorder; CGG: cytosine-guanine-guanine; CHC: Cattell-Horn-Carroll; CSS: change sensitive score; DSM-5: Diagnostic and Statistical Manual of Mental Disorders (fifth edition); EXIQ: extended Intelligence Quotient; FMR1: Fragile X Mental Retardation 1 gene; FMRP: fragile X mental retardation protein; FR: Fluid Reasoning; FSIQ: Full Scale Intelligence Quotient; FXS: fragile X syndrome; ID: intellectual disability; IQ: intelligence quotient; KN: Knowledge; MAE: mean age equivalent; MIND: Medical Investigation of Neurodevelopmental Disorders; NVFR: Von-verbal Fluid Reasoning subtest; NVIQ: Non-Verbal Intelligence Quotient; PPVT-3: Peabody Picture Vocabulary Test (third edition); QR: Quantitative Reasoning; Q-Q: quantile-quantile; RCPM: Raven’s Colored Progressive Matrices; SB5: Stanford Binet (fifth edition); VABC: Vineland Adaptive Behavior Composite; VABS-II: Vineland Adaptive Behavior Scales (second edition); VIQ: Verbal Intelligence Quotient; VS: Visual Spatial Processing; WISC: Wechsler Intelligence Scale for Children; WM: Working Memory.

## Competing interests

Dr. Hessl has received funding support from Roche, Novartis, and Seaside Therapeutics for consultation regarding clinical trials of patients with fragile X syndrome.

## Authors’ contribution

SS generated deviation scores, completed statistical analyses, participated in study design, and drafted the manuscript. EB organized, entered, and prepared IQ data at the UC Davis site. AS assessed many of the participants in the study. EBK provided FXS IQ data from the Rush University site and is the Rush PI. CP organized, entered, and prepared IQ data at the Rush site. DH conceived of the study, obtained permission and normative data from PRO-ED, Inc., obtained permission and data from the AGRE repository, directed and advised SS in the analysis of data and manuscript preparation, and wrote and edited portions of the manuscript. All authors read and approved the final manuscript.
